# Electrocardiographic screening in primary care for cardiovascular disease risk and atrial fibrillation

**DOI:** 10.1017/S1463423619000355

**Published:** 2019-06-25

**Authors:** Ralf E. Harskamp

**Affiliations:** Department of General Practice, Amsterdam University Medical Centers – Location Academic Medical Center, Amsterdam, The Netherlands

**Keywords:** ECG, electrocardiography, primary care, general practice, screening

## Abstract

Electrocardiograms (ECGs) are frequently recorded in primary care for screening purposes. An ECG is essential in diagnosing atrial fibrillation, and ECG abnormalities are associated with cardiovascular events. While recent studies show that ECGs adequately reclassify a proportion of patients based on the clinical risk score calculations, there are no data to support that this also results in improved health outcomes. When applied for screening for atrial fibrillation, more cases are found with routine care, but this would be undone when physicians would perform systematic pulse palpation. In most studies, the harms of routine ECG use (such as unnecessary diagnostic testing, emotional distress, increased health expenses) were poorly documented. As such, the routine performing of ECGs in asymptomatic primary care patients, whether it is for cardiovascular disease risk assessment or atrial fibrillation, cannot be recommended.

## Introduction

The electrocardiogram (ECG) has become a well-established diagnostic instrument in the field of (primary care) medicine. It can be used in a variety of clinical scenarios, such as chest pain, dyspnea, palpitations, and (pre)syncope. ECG is considered the gold standard for the diagnosis of cardiac arrhythmias and conduction disorders and for differentiating ST-segment elevation from non-ST-segment elevation acute coronary syndromes (Moyer *et al.* 2012; Goff *et al.* 2014). Apart from the evaluation of symptomatic patients, it is also commonly used for screening purposes in primary care. The rationale for performing routine ECGs in asymptomatic patients is to timely detect silent atrial fibrillation (AF) and/or to detect ECG abnormalities that are associated with coronary heart disease. Both conditions present a significant health burden, and coronary heart disease is a leading cause of death (Moyer *et al.* 2012; Goff *et al.* 2014). The rationale for ECG screening is supported by research that shows that ECG abnormalities, such as silent-Q-wave-infarctions, ST-segment and T-wave changes, left ventricular hypertrophy, and bundle branch blocks, are independently associated with future cardiovascular events and death, with hazard ratios ranging from 1.5 to 2.5 (Brown *et al.* 2000; Healy *et al.* 2016; Van der Ende *et al.* 2017). As such, ECG screening would appear an effective measure for preventing or timely indicating cardiovascular events. Certainly, an ECG can easily be performed, is low in direct cost, and covered by most healthcare insurance policies. The flipside, however, is that ECGs may be inconclusive or lead to false-positive results, in which unnecessary additional cardiovascular testing is performed. In turn, this leads to anxiety by the patient and increased healthcare spending without clear health benefits. This article provides a focused update on the currently available evidence on the delicate balance of harms and benefits of performing ECGs for screening purposes in primary care.

### Routine ECGs during annual physicals in low-risk individuals

In several countries, routine annual physicals or general medical examinations are a common form of preventive medicine in healthy adults. Resting ECGs are performed in approximately 20% of those visits. Bahia *et al.* (2017) reported the health effects of routine ECGs in a population-based study from Ontario, Canada. All 3.6 million study participants had an annual health examination by their primary care physician. All participants were found to have no known cardiovascular disease nor major cardiovascular risk factors. The authors subsequently compared healthcare utilization (cardiac tests, cardiology visits, cardiovascular procedure) and clinical outcomes between those who did and who did not receive a routine ECG. Overall, the authors found that the group who received a routine ECG were more likely to have cardiovascular follow-up or procedures (odds ratio: 5.14, confidence interval: 5.07–5.21). This did, however, not translate in a difference in clinical outcomes at 1 year, as measured by death (0.19% versus 0.16%), cardiac-related hospitalization (0.46% versus 0.12%), or coronary revascularization (0.20% versus 0.04%). It is clear that these data strongly oppose the routine use of ECGs during annual physicals in low-risk individuals.

### Integration of ECG to improve cardiovascular risk assessment

Significant effort has been undertaken to establish the role of routine ECGs as part of cardiovascular risk assessment in asymptomatic adults in the general population. A systematic review from Chou *et al.* (2011) summarized the available evidence up to 2011 (comprising data from more than 60 clinical studies). The authors found that abnormalities on resting ECG were associated with an increased risk for subsequent cardiovascular events after adjustment for traditional risk factors. However, no study compared clinical outcomes or use of risk-reducing therapies between persons who did and did not receive screening ECG. Also, no studies assessed whether ECG findings better classified patients into meaningful risk groups than did traditional risk factor assessment alone. This led the authors to conclude that the clinical implications of these findings were unclear.

Since the publication of this review study, there have been at least five population-based studies that have contributed to this discussion. One of the largest studies is the Health ABC population-based study of people aged 70 years or older in the United States (Auer *et al.* 2012). In this population, the addition of ECG abnormalities to conventional risk factors led to a 13.6% risk classification in intermediate-risk participants (overall net reclassification of 7.4%) over 4 years of follow-up. While ECG abnormalities were associated with improved risk prediction beyond traditional risk factors, the authors also showed that this also depended on which clinical prediction model was applied. In a Danish population study among people 65 years of age or older, the authors also found that including the ECG finding of left ventricular hypertrophy led to risk improvement compared with only the Heart Score and Framingham Risk score (Jorgensen *et al.* 2014). In a population study from the Netherlands, involving 2370 participants aged 38–74 years, the authors merely found a minor improvement in prediction for cardiovascular events in addition to conventional cardiovascular risk factors, in which the net reclassification improved 1% of future myocardial infarction and 0.5% of future AF (Goort *et al.* 2015). Recently, de Lemos *et al.* published the findings of a combined multi-modality risk assessment from two US-based population cohorts. In this study, they authors found that the ECG finding of left ventricular hypertrophy was one of the predictors that attributed to an improved clinical prediction score that led to reclassification of intermediate risk patients (de Lemos *et al.* 2017). Additional studies support this finding and suggest that regression of left ventricular hypertrophy could be achieved when underlying hypertension was adequately managed, resulting in an improved cardiovascular risk (Okin *et al.* 2004; Soliman *et al.* 2017; Harskamp *et al.* 2018).

In June 2018, the US Preventive Services Task Force published a large systematic review on the topic of ECG screening for cardiovascular disease risk, which included the findings of the aforementioned studies (Jonas *et al.* 2018a). The authors found that adding an ECG to traditional risk factors produced small improvements in discrimination and appropriate risk classification for prediction of multiple cardiovascular outcomes (based on nine cohort studies, *n* = 66,407). The total net reclassification improvements ranged from 3.6% (2.7% event, 0.6% nonevent) to 30% (17% event, 19% nonevent) for studies using clinical risk scores (ie, Framingham risk score). The authors, however, did find a number of important caveats that limited the quality of the data, most notably the considerable heterogeneity and a lack of quantification of the potential harms, from subsequent diagnostic work and/or revascularization. The overall recommendations based on these data can be found in Table 1.

**Table 1.** Recommendations on the use of a resting ECG as part of cardiovascular disease risk or screening for atrial fibrillation

Population	Recommendation
Adults at low risk of CVD events	Evidence against the use of ECG in this population: limited (if any) benefit, potential for harm
Adults at intermediate or high risk of CVD events	Better reclassification, unknown whether it leads to better clinical outcomes; insufficient evidence to assess the ratio of risk versus benefit
Older adults (>65 years of age)	No recommendation; insufficient evidence to assess whether screening with ECG identifies older adults with previously undiagnosed AF more effectively than usual care

ECG = electrocardiogram; CVD = cardiovascular disease; AF: atrial fibrillation

Hence, from a clinical perspective, what can we conclude from these data? Should we say that the jury is still out on whether an ECG improves cardiovascular risk assessment? The answer is not straightforward. In patients with low cardiovascular risk, the data are clear: routine use of ECGs for screening purposes should not be performed (Curry *et al.* 2018a). However, what to do in a majority of patients with intermediate and high cardiovascular risk? Better reclassification of these individuals by ECG could perhaps lead to more appropriate risk-modification interventions. The problem is that it has not been shown that such an approach does also lead to better clinical outcomes. In the setting of hypertension, an ECG can be informative on the presence of left ventricular hypertrophy, which can be used to more aggressively manage (uncontrolled) hypertension. For patients in whom the history is suggestive of past myocardial infarction, the finding of Q-waves is of importance. However, in a majority of patients, no general recommendation can be offered until clinical outcomes’ data become available. Moreover, ECG abnormalities often raise more questions than they answer, and an appropriate management or follow-up strategy is unclear. This dilemma is particularly eminent in ‘mild’ ECG abnormalities.

### Screening for AF

AF is a common cardiac arrhythmia, with a prevalence that is strongly correlated with age. The prevalence among young population is <1%, and it increases to ~4% in 60–70 year old people and 10–17% in those 80 years or older (Zoni-Berisso *et al.* 2014). Early diagnosis of AF is relevant to reduce the risks of developing heart failure, and more notably the risk of stroke. A recent systematic review including more than 135,300 patients from 17 studies showed that systematic screening in persons age 65 years and older results in an increase from 0.6% to 2.8% of AF cases found over a 12-month period (Jonas *et al.* 2018b). However, whether screening with ECG compared with no screening also led to improved health outcomes is not known and is the subject of the ongoing STROKESTOP trial (Svennberg *et al.* 2015). Moreover, two trials (*n* = 17,803) showed that systematic screening with ECG did not result in more AF cases when compared with a systematic approach using pulse palpation (Jonas *et al.* 2018b). In primary care, a number of randomized studies are underway to assess the role of (single or multiple-time point) handheld ECG devices, arrhythmia detection blood pressure machines, and pulse palpation (Uittenbogaart *et al.* 2015). The role of consumer-based single-lead ECG devices will also alter the landscape of screening for AF. An example is the ‘Apple Heart Study’ conducted in collaboration with Stanford (http://med.stanford.edu/appleheartstudy.html). While such ‘big data’ studies seem appealing, one should wonder whether these technology-driven studies represent the target population that we should focus on, and moreover whether there is appropriate awareness of the potential of harm when performing mass screening, which does not only include emotional distress but also the risk of misinterpretation of (single-lead) ECGs and subsequent unnecessary and potential harmful treatment (such as antithrombotic therapy for someone without AF). In summary, while there are many ongoing research efforts, there is currently a paucity of data in support of systematic ECG screening for AF. As such, at this time routinely ECG screening for AF cannot be recommended in primary care (Curry *et al.* 2018b).

## Conclusion

A resting ECG is of limited additional value in an asymptomatic general population. An ECG may reclassify patients when applied for cardiovascular risk assessment, but it is unclear whether adjusting risk management strategies also result in improved health outcomes. When applied for screening for AF, it results in more AF cases than routine care, but is similar when systematically performing pulse palpation. In most studies, the harms of ECG use have not been well documented. One should bear in mind the risk of false-positive ECG results, which results in more diagnostic testing with associated risks, induces anxiety and medicalization, and increased medical spending. This leads back to old but vital adage ‘primum non nocere’.
